# Evaluation of Some Plasma Coagulation Factors in Women with Spontaneous Miscarriage 

**DOI:** 10.22074/ijfs.2015.4545

**Published:** 2015-10-31

**Authors:** Mahsa Besharat, Afsane Tabandeh, Abbasali Keshtkar, Elham Mobasheri, Sima Besharat, Hamidreza Joshaghani

**Affiliations:** 1 Golestan Research Center of Gastroenterology and Hepatology, Golestan University of Medical Sciences, Gorgan, Iran; 2 Golestan Research Center for Infertility, Golestan University of Medical Sciences, Gorgan, Iran; 3 Endocrine and Metabolic Research Institute, Tehran University of Medical Sciences, Tehran, Iran; 4 Golestan Research Center of Congenital Abnormality, Golestan University of Medical Sciences, Gorgan, Iran; 5 Tehran University of Medical Sciences, Digestive Disease Research Institute, Golestan Research Center of Gastroenterology and Hepatology, Golestan University of Medical Sciences, Gorgan, Iran; 6 Medical Laboratory Research Center, Golestan University of Medical Sciences, Gorgan, Iran; 7 Department of Medical Laboratory Technology, School of Paramedicine, Building of Falsafi, Hircan Boulevard, Gorgan, Iran

**Keywords:** Thrombophilia, Abortion, Protein C, Protein S, Factor V Leiden

## Abstract

**Background:**

It has been reported that 15-20% of parous female have experienced at least one miscarriage, while 3% of them have experienced two miscarriages. The goal of this study was to evaluate the plasma level of coagulation factors in women with a history of spontaneous abortions.

**Materials and Methods:**

In this case-control study, 82 women with a history of two or more abortions referred to the six private gynecologic clinics in Gorgan city without any structural abnormality were recruited during 2011-2012. Plasma levels of antithrombin III (ATIII) using colorimetric assay, protein C, protein S, factor V Leiden and lupus anticoagulant (LAC) using coagulation method were measured. The control group was women with a history of normal delivery and no abortions. Those under anti-coagulant therapy were excluded from the study. Data were entered into the computer using the Statistical Package for the Social Sciences (SPSS, SPSS Inc., Chicago, IL, USA) version 16 and analyzed by Chi-square, t test and non-parametric tests.

**Results:**

At least one abnormality was reported in 35 cases (42.7%). Among them, protein C deficiency was the most prevalent (30.5%). ATIII was abnormal in 17.1% and lupus anti-coagulant was abnormal in 8.5%. Factor V Leiden was normal in all cases and protein S deficiency was only seen in one case.

**Conclusion:**

We suggest to perform these tests in regards to the thrombophilia in cases with spontaneous abortions in order to find an early cure for this treatable disorder.

## Introduction

It has been reported that 15-20% of all parous women have experienced at least one miscarriage, 3% have experienced two miscarriages and finally 1% have experienced three or more miscarriages (1). Recurrent pregnancy loss ( RPL ) is defined as two or more consecutive pregnancy losses before the 28^th^ week of pregnancy with fetus weighing less than 1000 g. RPL affects 2-4% of reproductive age couples worldwide(2,3). Several different etiologies have been defined for RPL such as: genetic factors, structural abnormalities, endocrine disorders like thyroid hormone alteration, immunologic factors, infective causes and unexplained causes (3). 

There are still debates on the role of immunologic factors as the pathogenesis of recurrent miscarriages (1,4). Several risk factors as definitive or probable causes of RPL have been reported for women with recurrent pregnancy loss, like acquired maternal thrombophilia and inherited thrombophilia that include factor V Leiden deficiency, activated protein C resistance, prothrombin G20210A and protein S deficiency (4,5). 

There are few data about the spontaneous pregnancy loss in Iranian women in regards to thrombophilia markers. Therefore, we conducted this study to evaluate the plasma level of thrombophilia markers in women with a history of two or more abortions. 

## Materials and Methods

In this case-control study, 100 women with pregnancy loss history were referred to the academic and six private gynecologic clinics in Gorgan city, during 2011-2012. The inclusion criteria were a history of at least two pregnancy losses and no structural abnormality in the genital tract. Those undergoing anti-coagulant therapy were excluded from the study. Considering the inclusion criteria, 82 women were enrolled in our study. This study was confirmed by the Ethics Committee of Golestan University of Medical Sciences, Gorgan, Golestan Province, Iran. After obtaining informed consent from participants, the checklist was filled and they were referred to the Immunohematology Laboratory of Paramedicine Faculty of Golestan University of Medical Sciences. Control group included 86 age-matched women who experienced a normal pregnancy history for less than a year. 

Plasma levels of anti-thrombin III ( ATIII ) was evaluated using colorimetric assay ( Stago Kit, Diagnostica Stago, Inc., France ). Protein C, protein S, factor V Leiden and lupus anticoagulant ( LAC ) were also evaluated using coagulation methods ( Hyphen Biomed Kit, Amelung coagulometer Instrument, France ). All laboratory tests were done in a single center. 

The lower limit of the reference interval values for protein C, protein S, ATIII and factor V Leiden are 70, 55, 80 and 1.8%, respectively. Reference interval value for LAC was normal, less than 40 seconds. 

Data were entered into the computer using the Statistical Package for the Social Sciences ( SPSS, SPSS Inc., Chicago, IL, USA ) version 16. Mean values of factors were reported. P value<0.05 was considered significant. 

## Results

Mean ( ±SD ) age was 28.97 ( ±5.19 ) years in case group and 29.18 ( ±5.93 ) years in control group. The median pregnancy loss in cases was 2 ( ±0.85 ), minimum 2 and maximum 7 abortions. In the control group, the minimum values belonging to protein C, protein S, ATIII and factor V Leiden were 70.1, 60, 80, and 1.9%, respectively, while maximum value for LAC was normal less than 39.8 seconds. 

Factor V Leiden was normal ( >1.8 ) in all cases. LAC was abnormal ( ≥40 ) in 7 ( 8.5% ), ATIII ( <80 ) in 14 ( 17.1% ), protein S ( <55% ) in 1 and protein C ( <70% ) in 25 ( 30.5% ) (Table 1). 

In about 35 cases ( 42.7% ), at least one abnormality was reported in the lab tests. No one had all abnormalities at once, but there was a case with triple abnormalities in protein C, ATIII and LAC. 

One case had both abnormalities in protein C and protein S. In 3 cases, there was an abnormality in both protein C and LAC, and in 8 cases, protein C and ATIII were abnormal at once. 

**Table 1:** Comparison of mean values of five thrombophilia factors between case and control groups


Factors	Case	Control	Pvalue

LAC(s)	34.1±14.7	32.0±6.5	0.323
ATIII(%)	111.4±52.1	123.1±52.3	0.152
FactorVLeiden (Ratio)	2.8±0.7	3.0±0.8	0.231
ProteinC(%)	90.4±38.6	119.6±35.7	<0.001
ProteinS(%)	105.2±23.7	102.0±20.2	0.353


All data were presented as mean ± standard deviation (SD). LAC; Lupus anticoagulant and ATIII; Anti-thrombin III.

## Discussion

Abnormality in protein C has been reported in a relatively high percent of the cases with a history of abortion in the present study. Abnormal Factor V Leiden and protein S were not seen almost at all, but in other studies, they were reported to have a high prevalence.

D’Uva et al. (6) from Italy reported thrombophilia in 78% of cases with history of recurrent abortion and 16% of controls. They reported that abnormal ATIII was not seen in their population. Furthermore their findings showed that Factor V Leiden heterozygosity was present in 5.2%, protein C deficiency in 1.7% and protein S deficiency in 13%, indicating that protein C and protein S levels are lower and higher, respectively, than what we found in our study group. 

Bellver et al. (5) from Spain designed a study on 4 groups including control group, without a history of abortion; unexplained infertility ( UI ) group, a history of fertility more than one year without any autoimmune endocrine diseases; *in vitro* fertilization ( IVF ) group; normal Caucasian women group aged under 38 years and with unsuccessful IVF; and recurrent abortion group. Combined thrombophilia ( abnormality in all five factors ) was seen in 3 ( 9.4% ) of controls, 3 of UI groups ( 9.7% ), 5 in IVF group ( 19.2% ) and 2 of recurrent abortions ( 6.7% ) . However, in the present, there was no case of combined abnormality. 

Mitic et al. (7) reported at least one congenital thrombophilia alteration in 54 ( 36.7% ) women with a history of abortion, while protein S deficiency was the most prevalent one among them. Our results showed that protein S deficiency is present in just one case and protein C deficiency is the most prevalent one. Our finding indicated that alteration in at least one factor was reported in 35 cases ( 42.7% ). 

Jyotsna et al. (8) from India reported a significantly lower protein C, protein S and ATIII in cases with a history of abortion. The level of protein C was lower than normal in 33.3% of their cases. Their findings showed that levels of protein C, protein S and ATIII were lower as compared to related values of our findings, while the percentage of abnormal tests are more than the present study. In a study by Saadati et al. (9) in Iran, 3 cases ( 8.4% ) and no control had LAC positive results. 

In a study performed in Pakistan, opposite results have been reported. Their study included 52 women with a history of recurrent miscarriage and 268 healthy controls. The values of protein factors C (5.7 in patients vs. 6.7% in the control group), protein S (3.8 in patients vs. 4.5% in the control group) and Leiden factor (19.2 in patients vs. 10.0% in the control group) were significantly different between patient and control groups. However, antithrombin deficiency in the control group was significantly greater than the patient group (1.9 in patients vs. 15.2 % in the control group) (10), suggesting that there may be a problem in their selection of subjects recruited into the control group.

## Conclusion

We suggested to perform these tests, specially protein C, in regards to the thrombophilia in cases with spontaneous abortions in order to find an early cure for this treatable disorder.
